# Dialysis-specific factors and incident atrial fibrillation in hemodialysis patients

**DOI:** 10.1080/0886022X.2020.1801467

**Published:** 2020-08-11

**Authors:** Seung Don Baek, Soomin Jeung, Jae-Young Kang, Ki Hyun Jeon

**Affiliations:** a Department of Internal Medicine, Division of Nephrology, Mediplex Sejong Hospital, Incheon, Korea; b Department of Internal Medicine, Division of Nephrology, Sejong General Hospital, Bucheon, Korea; c Department of Internal Medicine, Division of Cardiology, Mediplex Sejong Hospital, Incheon, Korea

**Keywords:** Atrial fibrillation, incidence, renal dialysis

## Abstract

**Background:**

Atrial fibrillation (AF) is common in end-stage renal disease patients. Besides the traditional risk factors, we aimed to find dialysis-specific factors for developing incident AF.

**Methods:**

From March 2017 to August 2018, we retrospectively reviewed all outpatient-based prevalent hemodialysis patients in our artificial kidney room, and they were followed up until August 2019. Dialysate calcium concentration (3 versus 2.5 mEq/L), time length (4 versus 3.5 h), frequency (thrice weekly versus twice weekly), dialyzer size (effective surface area of 1.4 m^2^ versus 1.8 m^2^), membrane permeability (high flux versus low flux), ultrafiltration rate (mL/kg/hour), and blood flow rate (mL/min) were evaluated.

**Results:**

Among a total of 84 patients, 15 (17.9%) had newly detected AF with a follow-up period of 21 (13.3–24) months. By performing multivariate Cox regression analysis, blood flow rate (mL/min) and ultrafiltration rate (mL/kg/h) were considered significant factors for developing incident AF (adjusted hazard ratio [HR], 0.977; *p* = 0.011 and adjusted HR, 1.176; *p* = 0.013, respectively), while dialysis bath, time length, and frequency, dialyzer size, and membrane type were not considered significant factors. Ultrafiltration cutoff rate of 8.6 mL/kg/h was the best predictive factor for incident AF (area under the curve-receiver operating characteristic [AUC-ROC], 0.746; *p* < 0.005), while blood flow rate was not considered a significant factor for incident AF in ROC analysis (AUC-ROC, 0.623; *p* = 0.126). Ultrafiltration rate was largely dependent on interdialytic weight gain (*p* < 0.005, linear-by-linear association).

**Conclusion:**

Higher ultrafiltration rate was associated with incident AF in hemodialysis patients.

## Introduction

Atrial fibrillation (AF) is common in end-stage renal disease patients. A previous study revealed that AF prevalence was up to 27% in long-term hemodialysis patients [1]. The morbidity and mortality associated with AF are substantial. Additionally, the treatment of AF in dialysis patients has caused a great deal of controversy, whether anticoagulation was performed or not, in this specific population [2].

Traditionally, age, hypertension, obesity, diabetes, and preexisting cardiovascular disease are well-known risk factors for the development of AF [3]. In dialysis patients, the above-mentioned factors are already prevalent because they are also considered the common risk factors in developing renal failure. Data regarding factors other than the traditional ones associated with developing AF, such as dialysis prescription specific, are scarce.

In a previous report, dialysis itself was regarded as a risk factor for AF [4], with volume and electrolyte shift being suggested as the possible mechanisms [5,6]. In the present study, we determined the dialysis-specific factors associated with developing incident AF. Various dialysis prescription-associated details were investigated. Accordingly, we believed that controlling dialysis prescription might reduce incident AF during hemodialysis.

## Materials and methods

### Study population

We retrospectively reviewed the medical records of 84 end-stage renal disease patients. All chronic maintenance, outpatient-based hemodialysis patients in Mediplex Sejong Hospital were included from March 2017 to August 2018 and were followed up until August 2019. All prevalent hemodialysis patients underwent dialysis at least 90 days prior to inclusion. The patients who were lost to follow-up, ended the study, or died were censored. There was one mortality case. The following was the exclusion criterion: the presence of previous history of AF. The study was approved by the local ethics committee (2019-087).

### Variables

Baseline demographic and clinical data were obtained, including age, gender, body mass index, dialysis vintage, comorbidity, vascular access type, location, and pre-dialysis blood pressure and heart rate. Dialysis prescription-related factors were the following: membrane permeability (high flux versus low flux), session frequency (thrice weekly versus twice weekly), time length per session (4 versus 3.5 h), dialyzer size (effective surface area of 1.8 m^2^ versus surface area of 1.4 m^2^), ultrafiltration rate (mL/kg/h), blood flow rate (mL/min), and dialysate calcium concentration (3 versus 2.5 mEq/L). High-flux dialyzer was defined as KUF (coefficient of ultrafiltration) >15 mL/mmHg or β2 microglobulin clearance >20 mL/min. Single-pool Kt/V and normalized protein catabolic rate were estimated every 3 months [7,8]. The ultrafiltration volume (pre-dialysis body weight – post-dialysis body weight) was summated over the first month after enrollment, and the mean ultrafiltration rate (mL/kg/h, ultrafiltration volume divided by the dialysis time length and dry body weight) was calculated. All variables were recorded at the time of enrollment.

### Hemodialysis prescription

In our hospital, routine hemodialysis prescription included 4 h per session, thrice weekly schedule with biocompatible membranes. Twice weekly, or three and half hour dialysis per session, was permitted to have incremental regimens in selected patients with acceptable residual renal function (estimated glomerular filtration rate >10 mL/min/1.73 m^2^) and individual preferences. Blood flow rate was maximally adjusted to achieve optimal dialysis adequacy and was limited according to the patients’ intolerance. Ultrafiltration rate was set by interdialytic weight gain and was adjusted according to the patients’ ultrafiltration rate tolerance. A calcium bath of 3 mEq/L was used initially, and a low calcium bath (2.5 mEq/L) was used when serum calcium level increased greater than 10 mg/dL. Dialyzer size was dependent on the patients’ body surface area (dialyzer surface area to body surface area ratio = 0.8–1.0). The type of membrane permeability was determined by dialysis adequacy to achieve optimal Kt/V (1.2 at a minimum).

### Atrial fibrillation

AF had to be diagnosed with either 12-lead or continuous electrocardiographic monitoring for >30 s. AF was considered paroxysmal in the case of spontaneous resolution, persistent when pharmacological or electrical cardioversion was needed, and permanent when it could not be terminated by any method [1]. Routine 12-lead electrocardiography was performed every 6 months, and continuous electrocardiography monitoring was performed during hemodialysis depending on the patients’ subjective symptom complaints or unstable vital signs.

### Statistical analysis

All data were expressed as mean ± standard deviation or median and interquartile ranges according to the distribution. Group difference was analyzed using a Mann–Whitney test. Pearson correlation and linear-by-linear association test were used to determine the relationship between dialysis and incident AF. All dialysis prescription-related factors were entered as covariates in a univariate Cox regression analysis. Subsequently, a multivariate analysis using identified factors that showed a statistical significance in a univariate analysis (*p* < 0.1) was performed to determine the independent factors associated with incident AF. Adjusted hazard ratios were calculated. Receiver operating characteristic (ROC) curve revealed the best cutoff for prediction using Youden’s index. Survival analysis in a multivariate Cox regression analysis was plotted between the cutoff. The number of patients at risk during follow-up was not applicable in the adjusted survival analysis. Statistical significance was set at *p* < 0.05. Statistical Package for the Social Sciences (SPSS) version 22.0 (SPSS, Chicago, IL, USA) and MedCalc version 14.8.1 (Mariakerke, Belgium) software were used for analysis.

## Results

### Baseline demographics and incident AF

A total of 84 patients were included in this study (Table 1). The mean age was 62.1 ± 12.7, 52 (61.9%) of the 84 patients were male, and the mean dialysis vintage was 20 (10–51.8) months. The average follow-up period was 21 (13.3–24) months.

**Table 1.** Baseline characteristics.

	No AF (*n* = 69)	AF (*n* = 15)	*p-*value
Follow-up (months)	20 (14–24)	24 (14.5–24)	0.334
Age (years)	61.4 ± 12.7	65.1 ± 12.4	0.317
Male	44 (63.8%)	8 (53.3%)	0.645
Body mass index (kg/m^2^)	23.1 ± 3.0	22.7 ± 3.9	0.631
Dialysis vintage (months)	18 (8–48)	27 (20.5–78)	0.064
Comorbidity			
Diabetes	36 (52.2%)	8 (53.3%)	1.000
Hypertension	65 (94.2%)	15 (100.0%)	0.774
Chronic glomerulonephritis	12 (17.4%)	2 (13.3%)	1.000
Coronary artery disease	23 (33.3%)	8 (53.3%)	0.246
Cerebrovascular disease	5 (7.2%)	1 (6.7%)	1.000
Vascular access type (native versus graft versus catheter)	48 (69.6%) versus 16 (23.2%) versus 5 (7.2%)	8 (53.3%) versus 6 (40.0%) versus 1 (6.7%)	0.402
Access location (excluding catheter) (upper arm versus lower arm)	8 (12.5%) versus 56 (87.5%)	2 (14.3%) versus 12 (85.7%)	1.000
Pre-dialysis systolic blood pressure (mmHg)	146.0 ± 21.4	145.9 ± 18.5	0.980
Pre-dialysis diastolic blood pressure (mmHg)	69.2 ± 15.0	64.9 ± 16.2	0.316
Pre-dialysis heart rate (rate/min)	70.5 ± 10.3	74.7 ± 13.0	0.176
Dialysis prescription			
Membrane (^a^high flux versus low flux)	8 (11.6%) versus 61 (88.4%)	0 (0.0%) versus15 (100.0%)	0.374
Thrice weekly versus twice weekly	62 (89.90%) versus 7 (10.0%)	13 (86.7%) versus 2 (13.3%)	1.000
Dialysis length (4 versus 3.5 h)	66 (95.7%) versus 3 (4.3%)	15 (100.0%) versus 0 (0.0%)	0.956
Dialyzer size (effective surface area of 1.8 m^2^ versus surface area of 1.4 m^2^)	7 (10.0%) versus 62 (95.70%)	0 (0.0%) versus 15 (100.0%)	0.439
Interdialytic weight gain (kg)	2.2 ± 0.9	3.3 ± 1.0	<0.001
Ultrafiltration rate (mL/kg/hour)	8.7 ± 3.2	11.6 ± 2.9	0.003
Normal calcium bath versus low calcium bath (3 versus 2.5 mEq/L)	55 (79.4%) versus 14 (20.6%)	14 (93.3%) versus 1 (6.7%)	0.358
Blood flow rate (mL/min)	250 (250–260)	250 (200–255)	0.116
Single-pool Kt/V	1.5 ± 0.2	1.4 ± 0.1	0.149
Normalized protein catabolic rate (g/kg/day)	0.9 (0.8–1)	0.9 (0.9–1.1)	0.334

^a^KUF (Coefficient of ultrafiltration) >15 mL/mmHg or β2 microglobulin clearance >20 mL/min.

A total of 15 patients (17.9%) experienced AF episode. Patients who experienced AF were categorized as follows: 10 patients (66.7%) had paroxysmal, 2 (13.3%) had persistent, and 3 (20%) had permanent AF. All paroxysmal episodes were detected during hemodialysis by continuous electrocardiogram monitoring and were spontaneously terminated during the hemodialysis day or before the next hemodialysis session. Two persistent AFs were terminated by diltiazem infusion. One of the three permanent AF episodes was detected by routine electrocardiogram screening without symptoms. Two of the three permanent AF patients underwent warfarinization.

### Different characteristics between incident AF and non-AF patients

There were no significant differences in comorbidity, follow-up period, vascular access type, location, pre-dialysis blood pressure, and heart rate, while there was an insignificant difference in dialysis vintage (*p* = 0.064).

**Figure 1.**
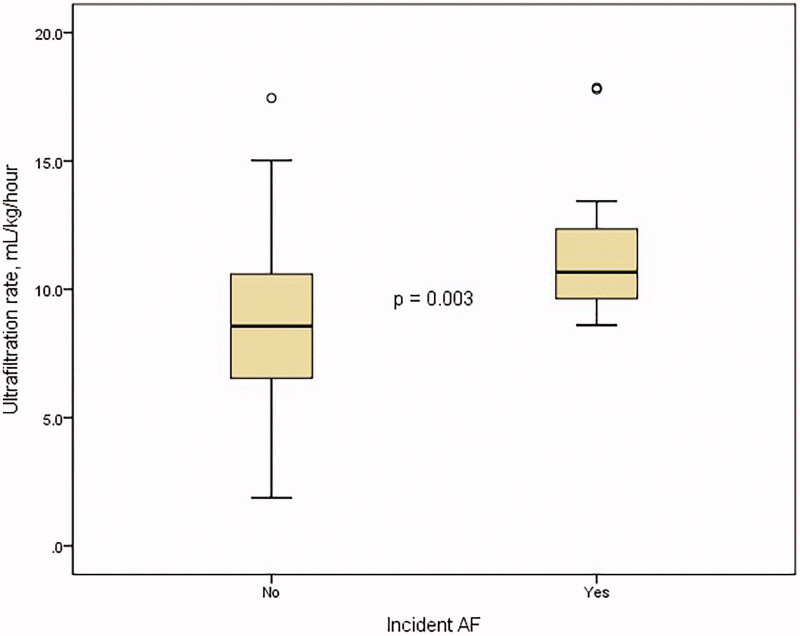
The difference in ultrafiltration rate between the groups.

**Figure 2.**
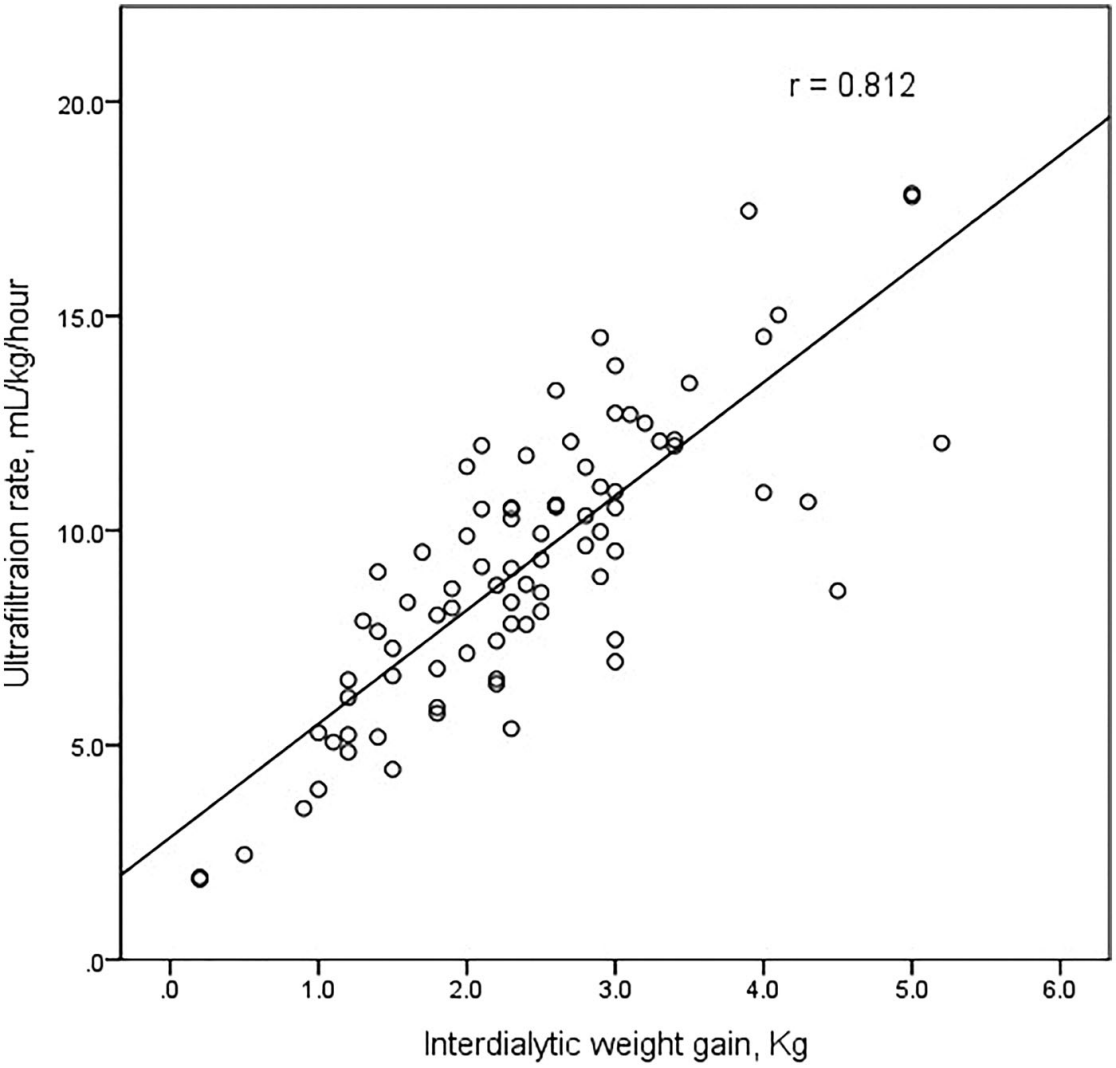
Scatter plot of the relationship between interdialytic weight gain and ultrafiltration rate.

Between the two groups, non-dialysis-related factors such as age, the prevalence of diabetes, hypertension, and coronary artery disease were comparable. However, interdialytic weight gain and ultrafiltration rate were significantly different (2.2 ± 0.9 kg versus 3.3 ± 1.0 kg, *p* < 0.005 and 8.7 ± 3.2 mL/kg/h versus 11.6 ± 2.9 mL/kg/h, *p* = 0.003, respectively, Figure 1). There is a significant correlation between interdialytic weight gain and ultrafiltration rate using linear-by-linear association (*p* < 0.005) and Pearson correlation (*r* = 0.812, *p* < 0.005, Figure 2). Membrane type, frequency, time length, dialyzer size, and dialysate calcium concentration were not significantly different.

Next, we determined the independent factors associated with incident AF. Among all the clinical variables, blood flow rate (mL/min) and ultrafiltration rate (mL/kg/h) were considered significant factors in a univariate Cox regression analysis (hazard ratio [HR], 0.977 (0.960–0.995); *p* = 0.011 and HR, 1.195 (1.041–1.372); *p* = 0.011, respectively). Moreover, two factors remained to be significant in a multivariate analysis (adjusted HR, 0.977 (0.959–0.995); *p* = 0.011 and adjusted HR, 1.176 (1.036–1.335); *p* = 0.013, respectively, Table 2).

**Table 2.** Variables associated with incident atrial fibrillation.

Variables	Adjusted hazard ratio (95% confidence interval)	*p-*value
Blood flow rate (mL/min)	0.977 (0.959–0.995)	0.011
Ultrafiltration rate (mL/kg/hour)	1.176 (1.036–1.335)	0.013

### Predictive performance of the identified factors and AF-free survival curve

**Figure 3.**
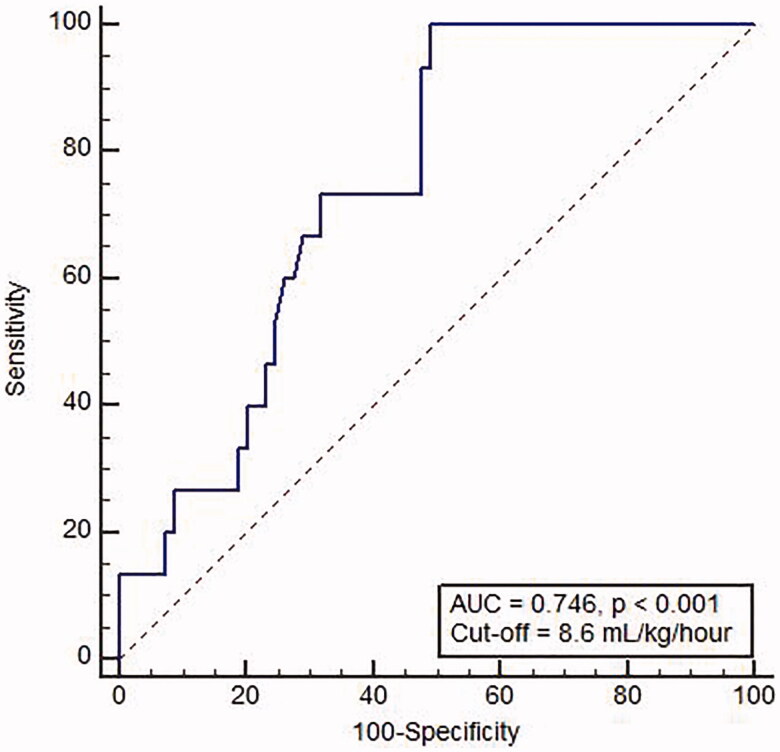
Receiver operating characteristic curve of ultrafiltration rate in predicting incident atrial fibrillation.

ROC curve of blood flow rate and ultrafiltration rate showed an area under the curve of 0.623 (0.511–0.727, *p* = 0.126) and 0.746 (0.639–0.835, *p* < 0.005), respectively (Figure 3). The best ultrafiltration cutoff of 8.6 mL/kg/h, which was considered a predictive factor of incident AF, showed a sensitivity of 100% and a specificity of 50.7%.

**Figure 4.**
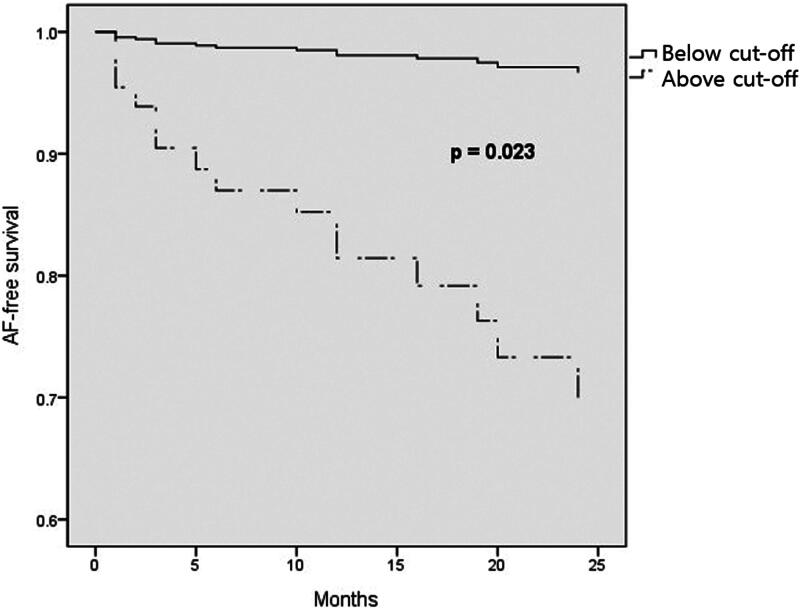
Adjusted survival analysis between above cutoff (≥8.6 mL/kg/h) and below cutoff ultrafiltration rates (<8.6 mL/kg/h).

We plotted the multivariate Cox regression survival curve divided by the ultrafiltration rate greater than 8.6 mL/kg/h and ultrafiltration rate less than 8.6 mL/kg/h. The curve showed a significant difference in AF-free survival between the two groups (*p* = 0.023) (Figure 4).

## Discussion

In this study, we showed that 17.9% of the maintenance dialysis patients experienced at least one episode of AF during less than 2-year follow-up period. All documented AFs were recorded during hemodialysis sessions, except one AF with routine electrocardiogram screening. Most of the AFs (66.7%) were paroxysmal, and 20% of patients had a permanent AF. Higher ultrafiltration rate was significantly associated with incident AF. We also demonstrated that ultrafiltration rate was generally dependent on interdialytic weight gain.

AF is more frequently observed in end-stage renal patients and leads to higher risk of stroke than that in the general population. Although AF could induce high morbidity and mortality in dialysis patients, its treatment is a matter of debate as prophylactic anticoagulation put those patients at higher risk of major bleeding compared to non-dialysis population [9]. The risk of hospitalization because of bleeding is also high [10]. The risk and benefit balance is difficult in dialysis patients. In this context, prevention and effort to identify the risk factors of AF other than the well-known conventional risk factors are warranted.

There is a shared etiology in AF development and chronic kidney disease occurrence [11]. Dialysis patients already had prevalent risk factors and were susceptible to AF. In our study, non-dialysis-related factors such as age, the prevalence of diabetes, and coronary artery disease were comparable between the groups.

Besides the traditional risk factors for incident AF, dialysis patients showed unique clinical features in developing AF. Hemodialysis itself induced intradialytic AF, which generally terminated without intervention after the dialysis session [12]. Continuous implantable cardioverter–defibrillator telemonitoring revealed that AF occurred more frequently during the dialysis day than during non-dialysis days. Similarly, we found that AF frequently occurred during hemodialysis. We also found that high ultrafiltration rate was associated with intradialytic AF development. Kim et al. reported that rapid ultrafiltration rate was associated with left atrial remodeling [13]. In that study, a cutoff of 10 mL/kg/h was considered a predictive factor of pathological increment in the left atrial volume index per year, which was comparable with our study. Even high ultrafiltration rate up to 13 mL/kg/h was associated with all-cause death in the Western population [14]. Left atrial size was significant not only in AF development but also in stroke, congestive heart failure, and cardiovascular death [15]. The left atrial volume was also an independent predictor of mortality in continuous ambulatory peritoneal dialysis patients [16]. It is assumed that a decreased intravascular volume by iatrogenic ultrafiltration affects atrial physiology and increases the risk of subsequent AF.

We also found that ultrafiltration volume was well correlated with interdialytic weight gain. Interdialytic weight gain is generally used as a basis for fluid and salt intake recommendation, and it guides the physicians in educating the patients about fluid and salt restriction [17]. Increased weight gain is associated with salt excess, high blood pressure, and left ventricular hypertrophy [18]. However, tighter control of volume expansion should be balanced with nutritional adequacy, especially in elderly patients. Obviously, the ultrafiltration rate is prescribed considerably based on interdialytic weight gain and patients’ maximal ultrafiltration tolerance. High ultrafiltration volume could induce muscle cramp, hypotension, and post-dialysis fatigue [19]. Dry weight could not be obtained with a single dialysis session in an overly hydrated state. For those patients, volume fluctuation between dialysis session by large fluid intake and high ultrafiltration rate could result in cardiovascular injury. A lower fluctuation of volume gain and loss caused less renin-angiotensin-aldosterone system activation and catecholamine release [20]. Benefits of extended hemodialysis with lengthier and more frequent sessions were suggested to reduce myocardial stress [21].

Among the dialysis-specific factors other than ultrafiltration rate, dialyzer size, membrane type, frequency, and time length were not considered as risk factors for incident AF. Although all of the above factors are associated with dialysis adequacy, they were not associated with incident AF. A lower dialysate calcium concentration was suggested to improve cardiovascular stability with increased risk of sudden cardiac death [22]. In our study, dialysate calcium concentration was not associated with incident AF. Lower blood flow rate was found to be a significant factor associated with AF in the regression analysis, but it was not a significant factor associated with AF in the receiver operating characteristic analysis. We believe that inevitable confounding factors might have led to the use of low blood flow rate at the cost of dialysis adequacy such as presence of a central catheter (7.1%, 6/84), poor fistula maturation, or preexisting heart failure [23]. Among the traditional risk factors, lower pre-dialysis blood pressure was associated with a high incidence of AF [24], with the included population older than those of our study. Inadequate ultrafiltration owing to low blood pressure and extracellular volume overload was a suggested mechanism.

This study has several limitations. First, this retrospective, small population study has an inherent bias. Second, AF was only detected by an electrocardiogram that was dependent on the patients’ complaints or unstable vital signs. An unrecognized paroxysmal AF episode might be detected during Holter monitoring or event recording, although electrocardiographic screening every 6 months was performed. Third, we did not investigate the echocardiographic parameters that could provide additional information such as the change in left atrial volume. The use of medication such as beta blockers or antiarrhythmic agents that could influence the incidence of atrial fibrillation was also missing.

In summary, we showed that ultrafiltration rate was a significant factor in developing AF in hemodialysis patients, irrespective of patient characteristics and underlying comorbidity. Higher ultrafiltration rate, which is dependent on higher interdialytic weight gain, with their association not surprising, is a physician-dependent order. Education to restrict fluid intake while maintaining nutritional adequacy is highlighted to prevent the occurrence of burdensome arrhythmia. Additionally, extended hemodialysis with a lower ultrafiltration rate might be considered in patients with high interdialytic weight gain.

## References

[1] Genovesi S, Pogliani D, Faini A, et al. Prevalence of atrial fibrillation and associated factors in a population of long-term hemodialysis patients. Am J Kidney Dis. 2005;46(5):897–902. 16253730 10.1053/j.ajkd.2005.07.044

[2] Lok CE. Paroxysmal atrial fibrillation in a patient on hemodialysis. Clin J Am Soc Nephrol. 2017;12(7):1176–1180. 28600457 10.2215/CJN.00790117 PMC5498343

[3] Watanabe H, Tanabe N, Watanabe T, et al. Metabolic syndrome and risk of development of atrial fibrillation: the Niigata preventive medicine study. Circulation. 2008;117(10):1255–1260. 18285562 10.1161/CIRCULATIONAHA.107.744466 PMC2637133

[4] Buiten MS, de Bie MK, Rotmans JI, et al. The dialysis procedure as a trigger for atrial fibrillation: new insights in the development of atrial fibrillation in dialysis patients. Heart. 2014;100(9):685–690. 24670418 10.1136/heartjnl-2013-305417

[5] Braunschweig F, Kjellstrom B, Soderhall M, et al. Dynamic changes in right ventricular pressures during haemodialysis recorded with an implantable haemodynamic monitor. Nephrol Dial Transplant. 2006;21(1):176–183. 16144845 10.1093/ndt/gfi145

[6] Zaza A. Serum potassium and arrhythmias. Europace. 2009;11(4):421–422. 19182234 10.1093/europace/eup005

[7] Daugirdas JT. Second generation logarithmic estimates of single-pool variable volume Kt/V: an analysis of error. J Am Soc Nephrol. 1993;4(5):1205–1213. 8305648 10.1681/ASN.V451205

[8] Depner TA, Daugirdas JT. Equations for normalized protein catabolic rate based on two-point modeling of hemodialysis urea kinetics. J Am Soc Nephrol. 1996;7(5):780–785. 8738814 10.1681/ASN.V75780

[9] Juma S, Thomson BK, Lok CE, et al. Warfarin use in hemodialysis patients with atrial fibrillation: decisions based on uncertainty. BMC Nephrol. 2013;14:174. 23941163 10.1186/1471-2369-14-174 PMC3751624

[10] Wang TK, Sathananthan J, Marshall M, et al. Relationships between anticoagulation, risk scores and adverse outcomes in dialysis patients with atrial fibrillation. Heart Lung Circ. 2016;25(3):243–249. 26481398 10.1016/j.hlc.2015.08.012

[11] Genovesi S, Vincenti A, Rossi E, et al. Atrial fibrillation and morbidity and mortality in a cohort of long-term hemodialysis patients. Am J Kidney Dis. 2008;51(2):255–262. 18215703 10.1053/j.ajkd.2007.10.034

[12] Harnett JD, Foley RN, Kent GM, et al. Congestive heart failure in dialysis patients: prevalence, incidence, prognosis and risk factors. Kidney Int. 1995;47(3):884–890. 7752588 10.1038/ki.1995.132

[13] Kim JK, Song YR, Park G, et al. Impact of rapid ultrafiltration rate on changes in the echocardiographic left atrial volume index in patients undergoing haemodialysis: a longitudinal observational study. BMJ Open. 2017;7(2):e013990. 10.1136/bmjopen-2016-013990 PMC5294025 28148536

[14] Flythe JE, Kimmel SE, Brunelli SM. Rapid fluid removal during dialysis is associated with cardiovascular morbidity and mortality. Kidney Int. 2011;79(2):250–257. 20927040 10.1038/ki.2010.383 PMC3091945

[15] Abhayaratna WP, Seward JB, Appleton CP, et al. Left atrial size: physiologic determinants and clinical applications. J Am Coll Cardiol. 2006;47(12):2357–2363. 16781359 10.1016/j.jacc.2006.02.048

[16] Kim SJ, Han SH, Park JT, et al. Left atrial volume is an independent predictor of mortality in CAPD patients. Nephrol Dial Transplant. 2011;26(11):3732–3739. 21430181 10.1093/ndt/gfr118

[17] Sarkar SR, Kotanko P, Levin NW. Interdialytic weight gain: implications in hemodialysis patients. Semin Dial. 2006;19(5):429–433. 16970745 10.1111/j.1525-139X.2006.00199_1.x

[18] Levin NW, Zhu F, Keen M. Interdialytic weight gain and dry weight. Blood Purif. 2001;19(2):217–221. 11150813 10.1159/000046944

[19] Chou JA, Kalantar-Zadeh K, Mathew AT. A brief review of intradialytic hypotension with a focus on survival. Semin Dial. 2017;30(6):473–480. 28661565 10.1111/sdi.12627 PMC5738929

[20] Agarwal R, Andersen MJ, Pratt JH. On the importance of pedal edema in hemodialysis patients. Clin J Am Soc Nephrol. 2008;3(1):153–158. 18057304 10.2215/CJN.03650807 PMC2390993

[21] Shafiee MA, Chamanian P, Shaker P, et al. The impact of hemodialysis frequency and duration on blood pressure management and quality of life in end-stage renal disease patients. Healthcare (Basel). 2017;5(3):52. 28869490 10.3390/healthcare5030052 PMC5618180

[22] Toussaint N, Cooney P, Kerr PG. Review of dialysate calcium concentration in hemodialysis. Hemodial Int. 2006;10(4):326–337. 17014507 10.1111/j.1542-4758.2006.00125.x

[23] Lee HS, Song YR, Kim JK, et al. Outcomes of vascular access in hemodialysis patients: analysis based on the Korean National Health Insurance Database from 2008 to 2016. Kidney Res Clin Pract. 2019;38(3):391–398. 31378011 10.23876/j.krcp.19.015 PMC6727887

[24] Chang TI, Liu S, Airy M, et al. Blood pressure and incident atrial fibrillation in older patients initiating hemodialysis. Clin J Am Soc Nephrol. 2019;4:1029–1038. 10.2215/CJN.13511118 PMC6625626 31175104

